# Lifetime Prevalence of Victimization and Perpetration as Related to Men’s Health: Clinical Insights

**DOI:** 10.3389/fpsyg.2022.762079

**Published:** 2022-03-15

**Authors:** Delia Leiding, Franziska Kaiser, Philippa Hüpen, Ramona Kirchhart, Andrei Alexandru Puiu, Marion Steffens, Rene Bergs, Ute Habel

**Affiliations:** ^1^Department of Psychiatry and Psychotherapy, Faculty of Medicine, RWTH Aachen University, Aachen, Germany; ^2^Center for Ambulant Psychotherapy, Röher Parkklinik, Eschweiler, Germany; ^3^GESINE Netzwerk Gesundheit.EN/Frauen helfen Frauen EN e.V., Schwelm, Germany; ^4^Center for Vocational Training, Berufsförderungswerk Düren GmbH, Düren, Germany; ^5^Jülich Aachen Research Alliance, Translation Brain Medicine, Jülich, Germany

**Keywords:** victimization, perpetration, victim-perpetrator, age-crime curve, health, adverse health behaviors, multiple violence

## Abstract

Violence is a known risk factor for health problems. In this epidemiological study across 5,385 male patients, we investigate the prevalence of perpetrated violence, exposure to violence, their overlap and the relationship between violence, mental, and psychosomatic health, as well as adverse health behaviors, such as self-harming behavior and the consumption of drugs. Participants completed an anonymous questionnaire addressing violence experience (i.e., both expose and perpetration), age of victimization/perpetration, frequency, and perceived severity of violence exposure. We considered physical, psychological as well as sexual violence. Information on health status and adverse health behaviors complemented the data. Results showed that 48.4% of the sample reported having experienced violence (perpetration, victimization, or both). The victim-perpetrator overlap formed the largest group, in which the incidence of having experienced multiple types of violence was significantly higher compared to victims and perpetrators. The age-crime curve flattened more slowly with increasing age in this group. Although the perceived severity of exposure to violence is lower in the overlap group, its health status and adverse health behaviors were worse. Interventions should focus on this group since they constitute a burden for the healthcare system.

## Introduction

Violence is an incisive event often associated with physical injuries and psychological stress posing increased health risks and resulting in high medical expenditures. There is currently no standardized and universal definition of violence nor is the assessment of violence homogenous. The heterogeneity in what is considered acceptable behavior is likely due to cultural influences, moral concepts, and social norms which are subject to continuous change. Nevertheless, the most accepted definition proposed by the WHO (Hoffman, 2003) states that violence is “the intentional use of physical force or power, threatened or actual, against oneself, another person, or against a group or community, that either results in or has a high likelihood of resulting in injury, death, psychological harm, maldevelopment, or deprivation.” Since both physical and psychological violence can have equally serious consequences (Pico-Alfonso et al., 2006), ongoing investigations should carefully look beyond the effects and consequences of physical violence (Imbusch, 2002). Our study examines a broad spectrum of physical, psychological, and sexual violence defined as all experiences of violent acts that were intentional and caused harm or had the potential to cause harm.

Victimization and perpetration of violence correlate well with each other and it is a well-documented finding that victims and perpetrators of violence are often the same people (e.g., Lauritsen and Laub, 2007; Jennings et al., 2010, 2012; Reingle and Maldonado-Molina, 2012; Beckley et al., 2017). Also in a sample of mentally ill patients, Johnson et al. (2016) identified that the group of victim-perpetrators is larger than the groups of victims only or perpetrators only. In a Caribbean sample, Debowska et al. (2021) showed that high rates of own youth victimization during adolescence formed strong correlations with attitudes toward violence (increased acceptance of physical violence). Moreover, youths who reported high/moderate levels of various types of violence were also more likely to perpetrate violent behavior (Debowska et al., 2018). Results of Leiding et al. (2021) in a clinical study add to these findings and showed that previous exposure to violence and the experience of multiple forms of violence (polyvictimization) predict perpetration. Thus, childhood victimization and attitudes toward violence may contribute to perpetration and/or re-victimization in adulthood. Related to intimate partner violence, Muftić et al. (2015) found that compared to victims (who were predominately female) and perpetrators (who were predominately male), victim-offenders were the most gender symmetric. Furthermore, research shows that perpetrators often commit multiple types of violence (polyperpetration) and that victims often experience multiple forms of violence (polyvictimization) (e.g., Beckley et al., 2017). Moreover, offenders tend not to concentrate their offending activities in specific offense types, but rather in multiple different types of offending (Piquero et al., 2012).

Regarding the incidence of violence, it is a well-known fact that criminal behavior is related to age. A typical “age-crime curve” (Farrington, 1986) shows an increase in violence prevalence from late childhood to late mid-/late adolescence where it peaks followed by a decrease thereafter [see also Piquero et al. (2007), Loeber et al. (2012), Moffitt (2018)]. Following a large nationally representative sample of adults (US National Comorbidity Study-Replication) over more than 9,000 participants, recent violence was more prevalent among younger individuals and males and violence generally decreased over one’s adult life (Fahlgren et al., 2020). However, violence perpetrators revealed no effect of age or gender on violence frequency (i.e., number of violent acts). This indicates that individuals who remain engaged in violence do so at relatively consistent levels (Fahlgren et al., 2020). Kazemian and Farrington (2018) showed that the residual career length and number of offenses remaining in criminal careers declines steadily with age. A similar age-crime-curve for victimization was also shown to peak during adolescence (Truman and Planty, 2012; Semenza et al., 2021) and a decrease thereafter. Even though older adults report violence at lowest rates, violence does exist even in late adulthood and is associated with major impact and health consequences (Rosen et al., 2019; Russo et al., 2019; Storey, 2020). Although, literature suggests that violence decreases over the life span there is also evidence that this is true for physical and sexual violence, but not for emotional abuse. According to a representative survey of over 10,000 German women, the prevalence of emotional abuse and controlling behavior remained nearly the same (Stöckl and Penhale, 2015) over the lifespan. Further, in advanced age additional forms of violence (e.g., caregiver abuse) and risk factors that are caused by age (e.g., higher dependency, vulnerability) exist (Biermann et al., 2011). However, to our knowledge, data on the relationship between age and violence experience in individuals who perpetrate and expose violent behavior (overlap group) is scare.

Another individual variable contributing to violence, in addition to age, is gender. Violence experiences (i.e., exposure as well as perpetration) occur in a variety of different areas and contexts in which both men and women may become victims and/or perpetrators. However, research on the relationship between gender and violence is less clear and often points to ambiguous results. Gender symmetry regarding rates of violence is a frequently and controversially discussed topic, which depends, at least in part, on the perspective and the type of data used (Straus, 2011). Indeed, gender symmetry is often supported on the basis of perpetration rates, whereas research that denies gender symmetry often argues based on the effects of victimization (Straus, 2011). For instance, Denson et al. (2018) reviewed that women tend to engage in intimate partner violence with equal frequency but lesser severity than men. Even though the proportion of male perpetrators seems to predominate crime statistics, the gender distribution varies depending on the context and type of violence (Bundesministerium für Familie, Senioren, Frauen. und Jugend, 2004; Schlack et al., 2013). There is evidence that prevalence rates of aggression are approximately equivalent across men and women in forensic samples (e.g., Smith and Waterman, 2006) and in clinical samples (e.g., Hamberger and Larsen, 2015; Habel et al., 2016; Douet et al., 2019). For example, Hamberger and Larsen (2015) conducted a review about intimate partner violence in clinical samples and found that, although both men and women participate in violent acts, their type and quality differ between sexes. In a previous German patient sample (Habel et al., 2016), 38% of all males and 43% of all females of 5,003 survey responders indicated one or more types of lifetime victimization experiences. While men experienced more physical violence in a public context (community violence that occurs in public places, perpetrated by strangers or distant acquaintances), women were more affected by psychological and sexual violence within their private social environment (domestic violence exerted by partners, family members, or friends) (Habel et al., 2016). A Swiss patient study supports these findings by showing that women and men experience violence equally, although women experience violence more often in a domestic context and men more often in a community context (Douet et al., 2019). However, gender-based violence rates seem not only be subjected to context and settings, but may also be influenced by cultural aspects. For example, in a study with a Caribbean sample, boys compared to girls reported increased levels of physical and sexual abuse in and outside the family (Debowska et al., 2018).

Yet, men’s victimization experiences receive less attention in research than women’s victimization experiences (Jungnitz, 2004). A balanced, comprehensive survey of male experiences of a wide range of different forms of violence, considering both victimhood and perpetration can therefore be of great importance when it comes to holistically investigating male experiences of violence and its relationship to health status and adverse health behavior. Both, a poor health status (mental as well as physical) as well as adverse health behaviors have societal consequences (e.g., care support, governmental actions) (Rivara et al., 2019). For example, posttraumatic stress disorder (PTSD), depression, anxiety disorder, and suicidal ideation were all linked to violence experiences (Sundaram et al., 2004; Pico-Alfonso et al., 2006; Reid et al., 2008; Moffitt et al., 2013; Rahtz et al., 2017; Newbury et al., 2018; Brunstein Klomek et al., 2019; Boduszek et al., 2021). A study of a non-Western sample found that sexual abuse can even lead to psychopathic personality traits (Boduszek et al., 2019). Among others, polyvictimization was found to be a high risk factor for mental health problems (Sabina and Straus, 2008; Ford et al., 2010). Individuals with repeated violence experience or multiple types of violence experience (polyvictimization) are particularly affected by health consequences (Hedtke et al., 2008; Ford et al., 2010; Brunstein Klomek et al., 2019). For instance, a review of 16 studies examining child abuse revealed that multiple victimization was associated with adverse internalizing and externalizing psychiatric outcomes (Debowska et al., 2017). Likewise, physical, psychological as well as sexual violence are associated with increased PTSD symptoms. Evidence points to a dose-response, where the more types of violence have been experienced, the more PTSD symptoms were present (Basile et al., 2004). However, not only mental health is linked to violence. There is also strong evidence supporting a link between (psycho)somatic diseases (e.g., Gini and Pozzoli, 2013), negative (chronic) physical health (e.g., Habel et al., 2016) consequences and violence. For example, gastrointestinal diseases, chronic pain syndrome, headaches, stomach ache, cardiovascular disease, inflammatory levels, and sexually transmitted infections (Kramer et al., 2004; Sundaram et al., 2004; Norman et al., 2012; Moffitt et al., 2013; Newbury et al., 2018; Rivara et al., 2019) are associated with violence.

It is not only the exposure to violence that is associated with poor health prognosis but also violence perpetration is related to health outcomes. Previous literature showed that violent offenders show poorer health status with high risk for chronic diseases, such as diabetes or migraine headaches (Reingle et al., 2014). Beyond the health status itself, adverse health behaviors have been identified in victims and perpetrators of violence. Self-harming behaviors have often been associated with violence (Walker et al., 2005; Newbury et al., 2018; Richmond-Rakerd et al., 2019; Boduszek et al., 2021). Moreover, a relationship between substance use and violence is well-established, whereby men in the victim-offender-overlap group had the highest levels of adverse health behaviors followed by those reporting perpetration only (Rhodes et al., 2009). Evidence therefore shows that people belonging to the victim-offender overlap group have a poor health prognosis and this may be partly because they suffer from the negative effects of both victimization and perpetration (Rivara et al., 2019). Health status and adverse health behaviors may have cumulative negative effects and the experience with violence can also contribute to the chronification of diseases and to delayed healing processes. The cause-and-effect relationship is by no means one-sided but rather a bi-directional interaction between violence and health. Nevertheless, mental illness is also a risk factor for violence [both victimization (Silver et al., 2005; Khalifeh et al., 2015) and perpetration (Van Dorn et al., 2012)]. It is therefore of utmost relevance to determine which health problems are associated with violence experience of different types of violence (Habel et al., 2016; Douet et al., 2019).

Violent behaviors exhibited by men appear more culturally accepted relative to violence perpetrated by women (Hagemann-White and Lenz, 2002). This, in turn, makes it difficult for men to recognize and accept male victimization. Furthermore, discrepancies in survey categories such as definition of terms, formulation of items, one-sided focus on physical violence or female victims continue to bias results (Jungnitz, 2004; Cho, 2012; Schlack et al., 2013). Therefore, in this study we focus on the relationship between different types of violence and men’s health outcomes. Looking at an only-male population, the prevalence questionnaire addresses perpetrated and exposed violence of male patients and their needs of support and care systems.

## The Current Study

This study aims to examine whether prevalence rates and results of previous studies can be replicated in a male patient sample. Therefore, we explore the prevalence of different types of violence, age of violence experiences, and polyvictimization as well as polyperpetration in a broadly defined male clinical population. Furthermore, we aim to identify group differences regarding health status and adverse health behaviors between four groups of interest (i.e., no-violence, victims only, perpetrators only, and victim-perpetrator-overlap). Based on previous literature we examine the following four tangible deliverables:

(1a) We hypothesize that the overlap group more often perpetrates multiple types of violence compared to perpetrators only.
(1b) We hypothesize that the overlap group is more often exposed to multiple types of violence compared to victims only.
(2a) Second, we hypothesize that exposure to violence peaks in adolescence and decreases thereafter in perpetrators (age-crime curve) and victims of violence (age-victim curve). Except for psychological violence, where we hypothesize that the age-victim curve remains stable over the lifespan as has been reported before in a sample of women.
(2b) In addition to our hypotheses regarding the age-crime curve of perpetrators only and the age-victim curve of victims only, we hypothesize that the age-curves in the overlap group peak in adolescence and decrease thereafter comparable to the age-crime and age-victim curves of perpetrators and victims only.
(3) Third, we hypothesize that the victims only and the overlap group display a greater number of health problems compared to the perpetrators only.
(4) Finally, we hypothesize that the perpetrators only and the overlap group display more adverse health behaviors compared to the victims only.

## Materials and Methods

### Participants and Procedure

This quantitative, epidemiological study included adult, male in- and outpatients from seven general and psychiatric hospitals in North Rhine-Westphalia, Germany. In the two city regions of the project partners, all hospitals were contacted and asked for permission to recruit there. Out of nine hospitals asked, seven gave their permission. Data were collected using an anonymous questionnaire on a voluntary basis. We excluded patients who were considered incapable (for example due to special capabilities, serious illnesses, or injuries) to understand the study information, making decisions and/or were not able to give or refuse to give consent. Participants were asked to complete the questionnaire without repercussions should they decide to exit the survey before completion. To ensure anonymity, participants received an unlabeled envelope for the completed questionnaire. The envelopes also ensured that participants could refuse anonymously to take part in this survey at any time. Participation was not remunerated. A total number of 5,385 male patients answered the questionnaire, 1,609 patients were not willing to fill out the questionnaire after oral clarification about the study (direct rejection) and 1,397 envelopes contained empty or incomplete questionnaires (indirect rejection). The rejection rate was 35.8%. Full details about the sample are reported elsewhere (Leiding et al., 2021). Participants received study information on two flyers. One provided further information about the project and study goals and the second offered contact details of a counseling service for victims and perpetrators of violence. Counseling was offered by a psychotherapist and was optionally available given the sensitive nature of the questions that could potentially trigger participants. This service was used by 38 individuals within the project duration. The Ethics Committee of the Medical Faculty of RWTH Aachen University was informed and provided with the material.

### Questionnaire Design and Data Collection

A structured and standardized questionnaire for the assessment of the prevalence of violence, which was developed in a previous study (Habel et al., 2016) was adapted and piloted on 106 patients for evaluation. The final questionnaire included 52 items for a differential assessment of victimization and perpetration in an open, half-open, multiple choice, and dichotomous answering format. The survey also includes rating scales. The final questionnaire was translated into five languages (German, English, French, Dutch, and Turkish) to reach as many participants as possible. The questionnaire was translated from the original language to the target language by a native speaker. A second independent translator then translated the questionnaire back into the original language. The original questionnaire and the back-translation were then compared for discrepancies. This procedure was repeated until both original versions were equal.

The questionnaire includes 12 items on demographic data such as age, number of children, highest level of education, marital status, occupation, and gross income per year. Violence-related questions were surveyed across three distinct categories including physical, psychological, and sexual violence (i.e., “Have you ever suffered physical abuse/suffered psychological abuse/experienced sexual violence?” “Have you ever physically/psychologically/sexually abused someone else?”). Following the violence research standards (Schröttle, 2016), accurate examples were listed for each type of violence. Mentioning specific violent incidents (e.g., hitting, mobbing, touching) may increase participants’ retrospective memory (Schröttle, 2016). Participants were asked to state all experiences of violent incidents which were intentional and caused harm or had the potential to cause harm. The age at which violence was experienced, information on the originator and target, the frequency of exposure and perceived severity (“How burdensome was the experience of violence for you? 0 not burdening—10 very burdening”) were recorded for each type of violence. In total, the experiences of violence were surveyed by 10 items for each category of violence. Additionally, the participants were asked about health-related complaints (e.g., “Which of the following illnesses or medical issues have had impact on your health?”) and adverse health behaviors (e.g., “Have you ever had dependencies or problems related to consumption of alcohol/drugs/sedatives/self-inflicted injuries/gambling/risky sexual activity …?”) using four items. Furthermore, the questionnaire includes six items on help-seeking behavior of the participants. For further information about development, piloting, and item details of this prevalence questionnaire, see Leiding et al. (2021).

### Statistical Analyses

We performed statistical analyses using SPSS statistics 25.0. Data were manually entered and 10% of all datasets were validated at random by a second independent researcher. A total of 5,385 datasets have been included in the analyses. The raw dataset was included in all analyses, with no type of imputation. Missing data were excluded from the respective analyses. Of the total sample, 784 participants (14.6%) had missing data on either exposed or perpetrated violence. Victims only, offenders only, and the victim-offender overlap group were compared regarding relative frequencies and mean values using chi squared- and *t*-tests. For health status and adverse health behaviors analyses we used binary logistic regressions. The occurrence of the respective health restriction or adverse health behavior is represented by the dependent variables with the coding 0 = no and 1 = yes. The categorically independent variable has the coding 0 = no violence, 1 = victims only, 2 = perpetrators only, and 3 = overlap, whereby “0 = no violence” was chosen as the reference category. Exp(beta) indicates how much the probability of a disease/adverse health behaviors increases or decreases in comparison to the reference category (odds ratio) (negative sign of the regression coefficients denotes an inverse relationship). All analyses were adjusted for the age of the participants. We applied the Bonferroni correction method for multiple comparisons.

## Results

### Sample Characteristics and Demographics

The sample characteristics of the participants that did not respond to questions on either violence exposure or perpetration and of those that did respond to all violence questions can be found in Supplementary Table 1 (Appendix A). Table 1 shows the demographic characteristics split per group: no violence experiences, neither victimization nor perpetration (none), victims only (V), perpetrators only (P), and (victim-perpetrator-)overlap (O).

**TABLE 1:** Sociodemographic characteristics of the groups (total *N* = 4,601)*.

	None (*n* = 2,376)	V (*n* = 855)	P (*n* = 222)	O (*n* = 1,148)

	N (Means ± SD) or (%)**			
Age (valid *n* = 4.601)	60.6 (±16.2) *n* = 2,376	53.3 (±17.1) *n* = 855	53.5 (±18.3) *n* = 222	47.9 (±17.8) *n* = 1,148
Country of birth *n* = 4,399, missing = 202	Valid *n* = 2,262, missing = 114	Valid *n* = 821, missing = 34	Valid *n* = 216, missing = 6	Valid *n* = 1,100, missing = 48
Germany	1,962 (82.6%)	727 (85.0%)	199 (89.6%)	974 (84.8%)
Other countries	300 (12.6%)	94 (11.0%)	17 (7.7%)	126 (11.0%)
Educational degree *n* = 4.539, missing = 62	Valid *n* = 2,343, missing = 33	Valid *n* = 841, missing = 14	Valid *n* = 220, missing = 2	Valid *n* = 1,135, missing = 13
No educational degree	35 (1.5%)	15 (1.8%)	4 (1.8%)	30 (2.6%)
Lower secondary school	938 (39.5%)	251 (29.4%)	86 (38.7%)	311 (27.1%)
Secondary school degree	486 (20.5%)	167 (19.5%)	40 (18.0%)	301 (26.2%)
European Baccalaureate	344 (14.5%)	163 (19.1%)	28 (12.6%)	241 (21.0%)
University degree	540 (22.7%)	245 (28.7%)	62 (27.9%)	252 (22.0%)
Marital status *n* = 4,424, missing = 177	Valid *n* = 2,297, missing = 79	Valid *n* = 817, missing = 38	Valid *n* = 216, missing = 6	Valid *n* = 1,094, missing = 54
Single	292 (12.7%)	179 (20.9%)	38 (17.1%)	344 (30.0%)
In a relationship	148 (6.4%)	94 (11.0%)	20 (9.0%)	110 (9.6%)
Married	1,547 (67.3%)	437 (51.1%)	133 (59.9%)	464 (40.4%)
Divorced/lived separated	185 (8.1%)	81 (9.5%)	19 (8.6%)	143 (12.5%)
Widowed	125 (5.4%)	26 (3.0%)	6 (2.7%)	33 (2.9%)
Employment status *n* = 4,311, missing = 290	Valid *n* = 2,242, missing = 134	Valid *n* = 773, missing = 82	Valid *n* = 216, missing = 6	Valid *n* = 1,080, missing = 68
Full-time	832 (35.0%)	339 (39.6%)	97 (43.7%)	459 (40.0%)
Part-time	53 (2.2%)	20 (2.3%)	8 (3.6%)	50 (4.4%)
Self-employment	122 (5.1%)	41 (4.8%)	11 (5.0%)	61 (5.3%)
Training courses	28 (1.2%)	22 (2.6%)	4 (1.8%)	40 (3.5%)
Retired	1,057 (44.5%)	290 (33.9%)	81 (36.5%)	267 (23.3%)
Unemployment benefits	75 (3.2%)	47 (5.5%)	5 (2.3%)	132 (11.5%)
Incapacitated for work	75 (3.2%)	36 (4.2%)	10 (4.5%)	71 (6.2%)
Annual gross income *n* = 3,565, missing = 1.036	Valid *n* = 1,804, missing = 572	Valid *n* = 667, missing = 188	Valid *n* = 171, missing = 51	Valid *n* = 923, missing = 225
0–10.000	151 (6.4%)	112 (13.1%)	14 (6.3%)	189 (16.5%)
10.000–20.000	341 (14.4%)	116 (13.6%)	29 (13.1%)	157 (13.7%)
20.000–40.000	682 (28.7%)	208 (24.3%)	61 (27.5%)	291 (25.3%)
40.000–60.000	370 (15.6%)	130 (15.2%)	47 (21.2%)	182 (15.9%)
>60.000	260 (10.9%)	101 (11.8%)	20 (9.0%)	104 (9.1%)

*Total sample was 5,385 of which 4,601 were included in the group analyses, since 784 participants had missing data on either exposed or perpetrated violence. **All percentages of total n of each group, including missing’s.

### Prevalence of Violence

51.6% of the participants who provided all information (*N* = 4,601) did not report any violence while 48.4% experienced violence throughout their life (be it victimization, perpetration, or both). Of all participants surveyed, 18.6% reported being victims of violence without having perpetrated violence themselves (abbreviated as V: victims only) and 25.0% reported being victims and perpetrators of violence (abbreviated as O: overlap). Only 4.8% reported having perpetrated violence without having experienced any type of violence exposure (abbreviated as P: perpetrators only).

**FIGURE 1:**
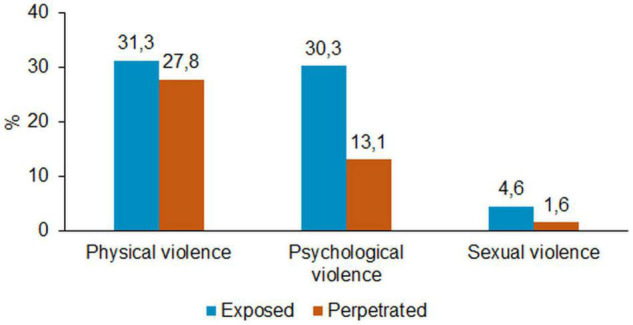
Types of exposed and perpetrated violence among all participants (multiple responses allowed).

Figure 1 shows the percentages of each violence category among all participants, excluding missing data for each variable. The exposure to physical and psychological violence is particularly notable. Almost one third of all participants were exposed to physical (31.3% of *N* = 5,075) and psychological (30.3% of *N* = 4,694) violence. The most frequent type of perpetrated violence was physical violence (27.8% of *N* = 4,338), followed by psychological violence (13.1% of *N* = 4,177). The victimization (4.6% of *N* = 4,669) and perpetration (1.6% of *N* = 4,143) of sexual violence was much less frequent.

### Victimization Perpetration Overlap

**FIGURE 2:**
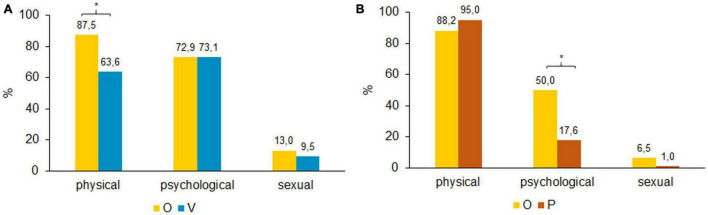
**(A)** Distribution of exposed violence among types of violence. Bonferroni corrected, *p < 0.05 (Multiple responses allowed). **(B)** Distribution of perpetrated violence among types of violence. Bonferroni corrected, *p < 0.05 (Multiple responses allowed).

Physical violence was the most frequent form of violence within the overlap group, whereas psychological violence was most often reported in the victim only group [Figure 2 shows the distribution of (A) exposed violence among victims only and overlap victims and (B) perpetrated violence among perpetrators only and overlap perpetrators]. The overlap group reported significantly more exposure to physical violence than the victim only group, *x*^2^(1) = 159.2, *p* < 0.000. There were no significant differences between these groups for psychological and sexual violence exposure.

Figure 2B shows that physical violence was the most frequent perpetrated type of violence for both groups (P and O), followed by psychological and sexual violence. The main difference was found for psychological violence, with 50.0% of the overlap group having exerted psychological violence compared to 17.6% among the perpetrators only group, *x*^2^(1) = 72.963, *p* < 0.000.

### Multiple Violence Experiences

**FIGURE 3:**
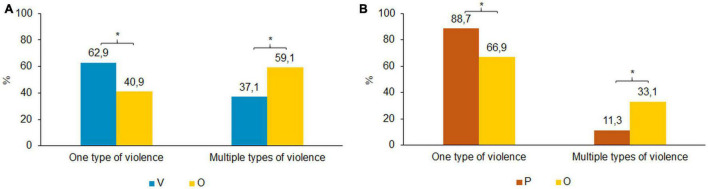
**(A)** One vs. multiple types of exposed violence. Bonferroni corrected, *p < 0.05. **(B)** One vs. multiple types of perpetrated violence. Bonferroni corrected, *p < 0.05.

A substantial number of participants experienced multiple types of violence as shown in Figure 3. In particular, the overlap group were exposed to polyvictimization, whereas the victims only experienced mostly one type of violence [*x*^2^(1) = 95.481, *p* < 0.000]. Although most perpetrators used one type of violence, about one third of the overlap group engaged in polyperpetration and about 11.0% of the perpetrators only exerted multiple types of violence. This difference was significant [*x*^2^(1) = 42.612, *p* < 0.000].

### Age of Violence Experiences

**FIGURE 4:**
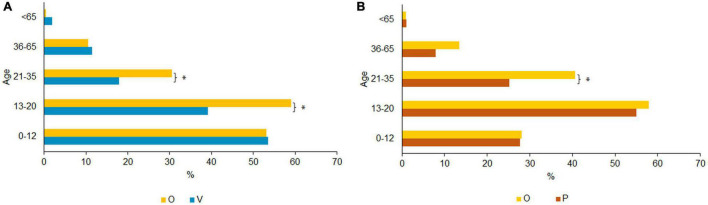
**(A)** Age of physical violence exposure. Bonferroni corrected, *p < 0.05 (Multiple responses allowed). **(B)** Age of physical perpetrated violence. Bonferroni corrected, *p < 0.05 (Multiple responses allowed).

Among the majority of the victims only group, physical violence took place between the ages of 0 and 12 (53.5%; Figure 4A). The majority of the overlap group, however, reported having experienced violence between the ages of 13–20 (59.0%). Differences between victims only and the overlap group are significant in the age range 13–20, *x*^2^(1) = 52.847, *p* < 0.000, as well as in the age range 21–35, *x*^2^(1) = 27.996, *p* < 0.000. Physical violence perpetration was most prevalent between the ages of 13 and 20 in all groups (O: 57.9% and P: 55.0%, Figure 4B). The overlap group used this type of violence significantly more often between the ages of 21 and 35 compared to the perpetrators only (O: 40.6% and P: 25.2%), *x*^2^(1) = 16.617, *p* < 0.000. There were no significant differences between the overlap and the perpetrators only groups for all other age categories. Victimization with respect to physical violence is most common in childhood, youth, and young adulthood. Perpetration of physical violence, on the other hand, is most common in youth/young adults after which it decreases after the age of 20 in parallel to physical violence experience.

**FIGURE 5:**
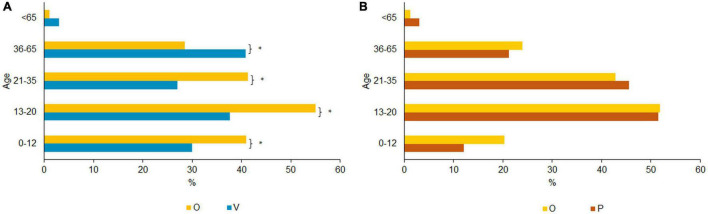
**(A)** Age of psychological violence exposure. Bonferroni corrected, *p < 0.05 (Multiple responses allowed). **(B)** Age of psychological perpetrated violence. Bonferroni corrected, *p < 0.05 (Multiple responses allowed).

As shown in Figure 5A the overlap group was exposed to psychological violence mostly between 13 and 20 years of age (55.0%). The V group mostly experienced psychological violence between 36 and 65 years (40.8%). Pairwise comparisons indicated significant differences between victims only and the overlap group. The overlap group was more frequently exposed to psychological violence than the victims only between 0 and 12 [*x*^2^(1) = 17.011, *p* < 0.000], between 13 and 20 [*x*^2^(1) = 40.009, *p* < 0.000], and between 21 and 35 [*x*^2^(1) = 29.428, *p* < 0.000]. Victims only were more frequently exposed to psychological violence between 36 and 65 years of age [*x*^2^(1) = 22.110, *p* < 0.000]. No significant differences emerged between the groups above the age of 65 with violence being rare after this age. The highest percentage of perpetrated psychological violence occurred between the age of 13 and 20 (O: 51.8% and P: 51.5%) and decreased with age (see Figure 5B). There were no significant differences between perpetrators only and the overlap group.

Due to the small sample size of victims (*n* = 77) and perpetrators only of sexual violence (*n* = 2) (particularly the perpetrators only), no valid statements can be made with regard to the age of sexual violence experiences.

### Estimation of Severity of Violence Exposure

**FIGURE 6:**
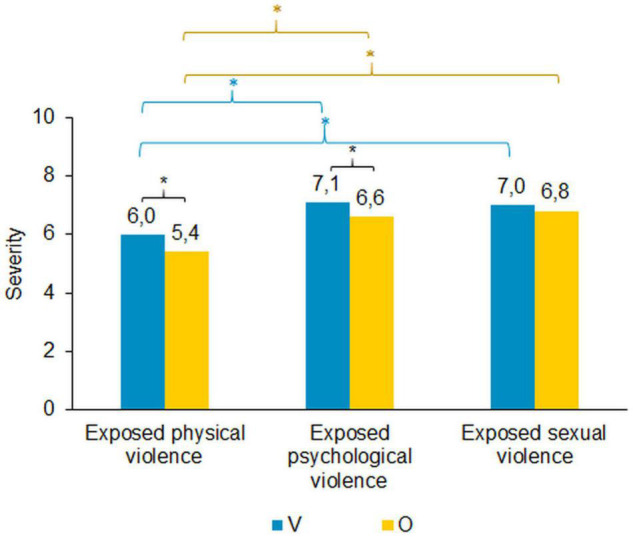
Estimated severity of the violence exposure, categorized by types of violence. *p < 0.05.

The subjectively perceived severity of exposure to violence was rated on a ten-point Likert scale, ranging from “not burdening” (0) to “very burdening” (10). Ratings for sexual and psychological violence were higher than for physical violence (Figure 6). These differences were significant for victims only {physical vs. psychological: [*t*(499) = −8.319, *p* < 0.000], physical vs. sexual: [*t*(499) = −7.582, *p* < 0.000]} as well as for the overlap group {physical vs. psychological: [*t*(957) = −11.749, *p* < 0.000], physical vs. sexual: [*t*(957) = −13.668, *p* < 0.000]}. No significant differences were found between perceived severity of psychological violence and sexual violence in both groups. The overlap group rated severity of physical violence [*t*(1456) = 3.415, *p* = 0.001] and psychological violence [*t*(1326) = 3.562, *p* < 0.000] significantly less severe than the victims only. There were no significant differences between groups in the perceived severity of sexual violence.

### Health Status

Table 2 shows the results of the binary logistic regression analyses regarding health status of the participants. All significant results that have an OR of at least 2 are marked in bold. The victims-only group has almost twice as high risk of being injured as the group that did not experience/exercise violence. The overlap group is almost twice as often affected. The likelihood of suffering chronic pain is also approximately double for the victims and perpetrators only, and twice and a half times for the overlap group. Overall, significant results are more often found in the victims only and the overlap group compared to the reference group. The probability of the occurrence of a disease is highest in the overlap group. This is particularly evident in the case of mental disorders (e.g., depression, anxiety, suicidal thoughts, PTSD). The probability of depression is roughly five times higher within the victims only and overlap group compared to the reference group. There is an approximately 4-fold risk of an anxiety disorder for these groups. PTSD is also significantly more likely to occur (V, OR: 6.9 and O, OR: 8.6) while the risk of suicidal thoughts is about 5 times higher within the victims only compared to the overlap group which is 7.2 times higher than the reference category (no violence). The victims only and the overlap group are particularly affected by negative health outcomes. This is especially true for mental disorders (depression, anxiety, suicidal thoughts, and PTSD) but also for sexually transmitted diseases or cardiac risks. The perpetrators only on the other hand have no or only slightly increased risk for most diseases.

**TABLE 2:** Health status.

Which of the following illnesses/medical issues have had impacts on your health?*	Variables in the equation	β	P**	Exp (β) (OR)
Injuries, broken bones (none 22.6%)	V (34.8%)	0.518	0.000	1.68
	P (33.5%)	0.506	n.s.	1.66
	O (40.7%)	0.660	0.000	1.93
Impairment of motor system (none 34.3%)	V (38.3%)	0.288	n.s.	1.33
	P (38.1%)	0.360	n.s.	1.43
	O (40.3%)	0.432	0.001	1.54
Permanent disabilities (none 11.3%)	V (13.5%)	0.326	n.s.	1.39
	P (10.6%)	0.117	n.s.	1.12
	O (13.1%)	0.436	0.000	1.55
Chronic pain (none 11.5%)	V (19.6%)	0.757	0.000	**2.13**
	P (17.9%)	0.723	0.000	**2.06**
	O (22.0%)	0.934	0.000	**2.55**
Gastrointestinal diseases (none 18.1%)	V (24.4%)	0.286	n.s.	1.33
	P (22.0%)	0.145	n.s.	1.16
	O (28.5%)	0.456	0.000	1.58
Respiratory diseases (e.g., asthma) (none 14.1%)	V (16.6%)	0.304	n.s.	1.36
	P (17.4%)	0.291	n.s.	1.34
	O (17.3%)	0.428	0.000	1.53
Skin diseases (e.g., neurodermatitis) (9.4%)	V (14.1%)	0.434	0.001	1.54
	P (9.7%)	0.045	n.s.	1.05
	O (13.6%)	0.394	n.s.	1.48
Sleep disorders (none 11.8%)	V (26.9%)	0.930	0.000	**2.53**
	P (16.1%)	0.238	n.s.	1.27
	O (29.4%)	0.996	0.000	**2.71**
Depression (none 7.0%)	V (30.6%)	1.590	0.000	**4.91**
	P (12.4%)	0.372	n.s.	1.45
	O (31.7%)	1.544	0.000	**4.68**
Anxiety (none 4.7%)	V (18.2%)	1.345	0.000	**3.84**
	P (5.0%)	−0.131	n.s.	0.88
	O (21.7%)	1.437	0.000	**4.21**
Heart Problems (none 31.7%)	V (29.4%)	0.160	n.s.	1.17
	P (29.4%)	0.224	n.s.	1.25
	O (23.4%)	0.107	n.s.	1.11
Suicidal thoughts (none 1.3%)	V (8.0%)	1.601	0.000	**4.96**
	P (3.7%)	0.869	n.s.	2.39
	O (12.6%)	1.979	0.000	**7.23**
Post-traumatic stress disorder (PTSD) (none 0.8%)	V (5.2%)	1.937	0.000	**6.94**
	P (1.8%)	0.893	n.s.	2.44
	O (7.1%)	2.155	0.000	**8.63**
Gender-related pathologies (e.g., prostate gland) (none 15.1%)	V (14.6%)	0.318	n.s.	1.37
	P (13.8%)	0.162	n.s.	1.18
	O (12.5%)	0.363	n.s.	1.44
Sexually transmitted diseases (STDs e.g., syphilis) (none 0.2%)	V (1.0%)	1.467	n.s.	4.34
	P (0.0%)	−15.197	n.s.	0.00
	O (1.1%)	1.310	n.s.	3.70
None of the aforementioned (none 9.5%)	V (5.2%)	−0.790	0.000	0.45
	P (4.1%)	−1.174	n.s.	0.31
	O (5.1%)	−0.908	0.000	0.40

*Reference category: none (no exposure to violence and no perpetration of violence). **Adjusted p after correction for multiple comparisons (Bonferroni) = 0.0016. All significant results that have an OR of at least 2 are marked in bold.

Furthermore, we created higher-order composite scores to investigate general health and violence. Please see Appendix B in Supplementary Material.

### Adverse Health Behaviors

Table 3 shows the results of the binary logistic regression analyses regarding adverse health behaviors. An increased likelihood to exhibit adverse health behaviors occurred especially for the group of victims only and the overlap group. With the exception of an increased probability of using alcohol, cannabis, and cocaine, perpetrators do not have an increased probability for the surveyed risk-taking behaviors. The overlap group showed the highest odd ratios for all risk-taking behaviors compared to the reference group. Particularly noteworthy is the increased probability of self-injury (12.5 times for the victims only and 20.5 times for the overlap group), as well substance abuse (i.e., hard and prescription drugs).

**TABLE 3:** Adverse health behaviors.

Have you ever had dependencies or problems (including sporadic incidents) related to:*	Variables in the equation	β	P**	Exp (β) (OR)
Smoking or other use of nicotine (none 59.4%)	V (64.1%)	0.251	n.s.	1.29
	P (69.3%)	0.459	n.s.	1.58
	O (71.9%)	0.626	0.000	1.87
Consumption of alcohol (none 45.5%)	V (51.0%)	0.242	n.s.	1.27
	P (56.6%)	0.506	0.001	1.66
	O (64.2%)	0.823	0.000	**2.28**
Increased sexual desire (none 6.4%)	V (15.4%)	0.874	0.000	**2.40**
	P (12.3%)	0.590	n.s.	1.81
	O (22.9%)	1.242	0.000	**3.46**
Risky sexual behavior (e.g., without condoms, frequently alternating partners) (none 2.4%)	V (6.0%)	0.860	0.000	**2.36**
	P (5.7%)	0.656	n.s.	1.93
	O (13.7%)	1.548	0.000	**4.70**
Self-inflicted injuries (none 0.2%)	V (3.4%)	2.528	0.000	**12.524**
	P (0.5%)	0.441	n.s.	1.55
	O (7.6%)	3.020	0.000	**20.481**
Sedatives (e.g., benzodiazepines) (none 4.7%)	V (13.0%)	1.082	0.000	**2.95**
	P (6.1%)	0.168	n.s.	1.18
	O (18.2%)	1.400	0.000	**4.06**
Consumption of psychedelic drugs (e.g., magic mushrooms) (none 0.7%)	V (2.1%)	0.709	n.s.	2.03
	P (2.4%)	0.605	n.s.	1.83
	O (6.3%)	1.503	0.000	**4.50**
Consumption of cannabis (none 4.7%)	V (15.6%)	1.028	0.000	**2.80**
	P (14.2%)	0.909	0.000	**2.48**
	O (27.9%)	1.546	0.000	**4.69**
Consumption of crystal meth (none 0.0%)	V (0.4%)	1.635	n.s.	5.129
	P (0.0%)	−13.977	n.s.	0.00
	O (1.0%)	2.314	n.s.	10.12
Consumption of ecstasy (none 0.9%)	V (2.9%)	0.763	n.s.	2.14
	P (4.7%)	1.207	n.s.	3.34
	O (8.4%)	1.542	0.000	**4.68**
Consumption of cocaine (none 1.0%)	V (3.9%)	1.099	0.000	**3.00**
	P (5.2%)	1.485	0.000	**4.41**
	O (9.7%)	1.802	0.000	**6.06**
Consumption of heroine (none 0.1%)	V (1.4%)	2.174	0.000	**8.79**
	P (0.0%)	−14.829	n.s.	0.000
	O (2.5%)	2.511	0.000	**12.32**
Prescription drug abuse (none 0.4%)	V (2.5%)	1.536	0.000	**4.65**
	P (0.0%)	−15.945	n.s	0.000
	O (6.6%)	2.350	0.000	**10.49**
Gambling (none 2.0%)	V (4.3%)	0.472	n.s.	1.60
	P (5.3%)	0.790	n.s.	2.20
	O (10.7%)	1.266	0.000	**3.55**
None of the aforementioned (none 24.5%)	V (20.9%)	−0.235	n.s.	0.79
	P (17.9%)	−0.412	n.s.	0.66
	O (11.6%)	−0.842	0.000	0.43

*Reference category: none (no exposure to violence and no perpetration of violence). **Adjusted p after correction for multiple comparisons (Bonferroni) = 0.0016. All significant results that have an OR of at least 2 are marked in bold.

Furthermore, we created higher-order composite scores to be able to make statements about general adverse health behaviors and violence.

## Discussion

This study explored the prevalence rates of different types of violence, the overlap between victimization and perpetration (1), the age-crime and age-victim curves (2), and the perceived severity of experienced violence. We additionally investigated whether the groups differed concerning their health status (3), and adverse health behaviors (4).

(1) Our results show a high prevalence of 48.4% of experienced violence (victimization, perpetration, or both) in this male patient sample. Overall, 43.6% (18.6% Victims only and 25% victim-perpetrators) of men in our sample experienced violent victimization, a prevalence nearly equal to that shown in a study by Plichta and Falik (2001) in which 43.7% of the 1,821 women included experienced victimization. Regarding the perpetration of violence, previous research found gender differences by showing that men were more likely to engage in violence, but this effect was moderated by age. In late adolescence (18–24) and older age (65+) women were as likely as men to report violence (Fahlgren et al., 2020). The victim-perpetrator overlap forms the largest group in our sample with a quarter of all participants reporting victimization and perpetration. The incidence of multiple violence was significantly higher in the overlap group. Results show that this group more often reported having been exposed to polyvictimization compared to victims only. Additionally, the overlap group more often reported having perpetrated multiple types of violence compared to perpetrators only. These high prevalence rates of violence and the large overlap between victimization and perpetration are in line with previous findings (Mustaine and Tewksbury, 2000; Lauritsen and Laub, 2007; Jennings et al., 2010, 2012; Silver et al., 2011; Beckley et al., 2017). The majority of the participants who had experienced violence identified as victim-perpetrator overlap (25.0%), followed by 18.6% victims only. Perpetrators only accounted for 4.8%. These findings are in accordance with results of a study among adults with mental illnesses [Johnson et al. (2016) that found a similar distribution of 18.7% victim-perpetrators, 13.2% victims only, and 5.3% perpetrators only]. A potential explanation could be that low self-control among men may account for a substantial portion of the victim-offender overlap (e.g., Flexon et al., 2016; Reisig and Holtfreter, 2018). Low self-control could also explain why this group exhibits the most risky behaviors, as it has been shown that people with low self-control are likely to take higher risks (e.g., Friehe and Schildberg-Hörisch, 2017). Alternatively, it may be that some individuals with early childhood experiences of victimization adopted maladaptive behavioral aggressive strategies for their own assertiveness later in life predisposing them to engage in violence throughout later in life (e.g., Voith et al., 2017). For instance, physical or sexual abuse during childhood are strong predictors for violence later in life that could either take form of victimization and/or perpetration. Emotional abuse, on the other hand, appears to best predict victimization and not perpetration (Voith et al., 2017). Our findings support this argument as we show that men who became victims only reported more psychological violence than men of the overlap group. Moreover, results reveal that, while the victim-only group was more often affected by psychological violence, the victim-perpetrators group was more often subjected to physical violence. The overlap group, however, exerted more often psychological violence compared to the perpetrator group only. Victims of the overlap group rated the perceived violence severity lower than victims only. Rating the perceived severity of violence lower could be due to internally and externally oriented processes, namely self-protection of negative violence effects as well as vindicating own actions and strategies of compensation and therefore normalizing acts of violence (Hagemann-White, 2006). It is possible that violent behavior is used to compensate own experiences of violence.
(2) The results partly support our second hypothesis. For physical violence, our results corroborate the age-crime curve proposed in previous research (Farrington, 1986; Piquero et al., 2007; Loeber et al., 2012; Truman and Planty, 2012; DeCamp and Zaykowski, 2015; Moffitt, 2018) and show a peak between the ages of 13 and 20 and a downslope curve with increasing age independent of violence exposure or use. The overlap group was more often involved in violence (compared to the victim group) between the ages of 13 and 35. Our findings extend the existing literature and show that the curve flattens slower in the overlap group compared to perpetrators only. Between 21 and 35 years, the overlap group is still more likely to use violence than the perpetrators only are. However, the results should be interpreted with caution since there is little preliminary evidence on the relationship between age-crime/age-victim curves and the victim-perpetrator overlap. Further research is needed to verify these results. Psychological violence, however, is not completely following the typical age-crime curve in our data. There are indications that prevalence rates of emotional abuse of women are roughly consistent with increasing age (Stöckl and Penhale, 2015). We do not find consistent violence rates across all age groups, but instead, exposure to psychological violence shows a second peak between 36 and 65 years for victims only but not for the overlap group. These findings may likely reflect “bullying at work,” as a form of psychological violence which takes place after childhood and youth (results of our data, not reported in this manuscript). Psychological violence was mostly experienced by colleagues in our data. This form of violence seems to affect victims only more often than the victim-perpetrator group [see Supplementary Figure 3 (Appendix C)].

To explore the third (3) and fourth (4) hypothesis, we compared three groups (V, P, and O) to participants with no violence experiences concerning their health status and adverse health behaviors. Overall, the overlap group exhibit the worst health status and the highest likelihood for adverse health behaviors (even though they rated the perceived severity of violence exposure lower than the victims only). The victim-group showed particular high numbers of negative health outcomes and adverse health behaviors. The negative health outcomes are mostly related to mental health concerns.

A particularly increased risk exists in the groups of victims only and the overlap group for chronic pain, sleep disorders, depression, anxiety, suicidal thoughts, and PTSD. Regarding adverse health behaviors the risks for risky sexual behaviors, self-injurious, consumption of hard drugs, and sedatives are particularly increased in the victim only and overlap groups. Rhodes et al. (2009) also found that men who reported victimization and perpetration had the highest frequencies of negative health outcomes as well as rates for smoking, alcohol abuse, and drug use. Negative effects of victimization and perpetration tend to co-occur in the overlap group which may constitute a rationale for the high odds ratios for most of the health conditions and adverse health behaviors. Rivara et al. (2019) already pointed out that victim-perpetrators suffer from negative effects of victimization as well as negative effects of perpetration. The fact that the group of none violence is significantly younger than the other groups and still has the best health status confirms our results, that the health problems of the other groups (mainly V and O) are probably due to the experience with violence. The use of drugs is a well-known risk factor for involvement in violence (e.g., Ahonen et al., 2019). Specifically, situational and behavioral risk factors such as being intoxicated could explain victimization in those who experience victimization only (Berg and Felson, 2016). Furthermore, this may be paired with offenders’ tendencies to be impulsive and short-sighted (Berg and Mulford, 2017), thereby increasing the likelihood of attacking someone of superior power which also heightens the risk for own victimization (Archer and Benson, 2008; Berg and Felson, 2016). Although the association between risky behaviors and violence is strong, there is no simple causal relationship, but complex pathways and interactions. Behavior and violence are probably mutually dependent and reinforcing in a vicious circle. Regarding health status, Ahonen et al. (2019) found that the attributable risk of mental illness to explain violence is low and most mental health problems are not independent predictors of violence when accounting for substance use or previous violence. However, this causal relationship is far from being clear.

Commonly found health effects and adverse behaviors in female victims of violence, such as chronic pain, sleep problems, suicidal thoughts, anxiety, depression, posttraumatic stress disorder, and substance abuse (Chrisler and Ferguson, 2006) were also identified in our male sample but with the highest incidence in the overlap group. However, previous research directly comparing male and female victims of violence suggests that there are qualitative rather than quantitative differences in health outcomes. A previous study by Habel et al. (2016) comparing male and female patients who had experienced violence found that male patients were at increased risk for adverse health behaviors (such as alcohol, drug, and nicotine use) compared to women. Conversely, a higher risk for chronic pain and self-injurious behavior was found in female patients. This pattern corresponds to known gender-specific patterns of behavior in the contest of mental disorders, i.e., externalizing behavior is frequently described in men and internalizing behavior in women (Habel et al., 2016). The present study extends these results, by surveying not only victimization but also perpetration of violence. Results of the present study indicate that the overlap group exhibit the worst health status and the highest likelihood for adverse health behaviors. For instance, an increased risk for gastrointestinal diseases (which is also often found in female victims, e.g., Chrisler and Ferguson, 2006), could only be found in the overlap group but not in victims only in our sample. Further research is needed to examine gender differences when comparing not only the consequences of victimization but also of perpetration and especially of the victim-perpetrator overlap.

Overall, the results suggest that most men who get in contact with violence tend to experience victimization and perpetration. Those participants has had the longest contact with (multiple) violence, since the age-crime and age-victim curves flatten more slowly in the overlap group. Polyvictimization and polyperpetration are characteristic for this group. Furthermore, this group has the poorest health status and shows the most adverse health behaviors. Although the perceived severity of the experienced violence is lower in the overlap group, they still report the worst health status and the highest likelihood for adverse health behaviors. Therefore, the overlap group represents a group with a particularly high risk of poor health prognosis regardless of the exact causes. To explore the causal relationship, longitudinal research is needed.

### Limitations and Strengths

Our results should be considered in the light of several limitations. First, due to the small sample size of the victims only (*n* = 77) and perpetrators of sexual violence (*n* = 2) groups, we defer making any statements regarding the age-crime- and age-victimization-curve of sexual violence likely owing to sex offending being relatively rare. Analyses about the age distribution of sexual violence should rather be made by future research that focus on sexual violence and therefore have a larger sample size. Second, the data were surveyed *via* self-reports. This method has the advantage that not only officially recorded crimes are taken into account. Due to this and the anonymity of the study, it may be assumed that the actual frequency and the age at onset of violence will be uncovered to a greater and earlier extent compared to official crime records. However, it should be noted that despite a high level of agreement between self-reports and official arrest reports (Dubow et al., 2014; Gomes et al., 2018), we cannot rule out that the data may be influenced by social desirability and memory biases. To avoid socially desirable answering, the survey was fully anonymous and we listed accurate examples for each violence type to increase participants’ retrospective memory. However, Farrington et al. (2014) concluded that the gap between self-reported violence and official records may even provide an opportunity for early intervention. Third, we cannot determine that the health outcome and adverse health behaviors are direct causal consequences of violence experiences. Our data solely demonstrate the distribution of the health status and adverse health behaviors between the different groups. Nevertheless, our results emphasize that the overlap group is particularly vulnerable. Although we cannot draw causal inferences given the study’s design, our results provide guidance for future longitudinal research and highlights potential groups that would benefit for intervention strategies. Last, completing the survey was accompanied by a certain time requirement due to the length of the questionnaire and a topic with negative valence. When collecting surveys, some respondents expressed being more informed about violence in general, mostly regarding the perception and boundaries between problematic and non-problematic behavior which the questionnaire clearly defined.

A notable strength of this study is the large sample size of patients from seven different hospitals. Nevertheless, it should be noted that the study sample was recruited from the region of North Rhine-Westphalia, Germany. Hospitals were selected based on the researchers’ location and therefore there was no randomization of hospitals. Therefore, potential biases cannot be fully excluded and the generalizability of our results might be limited. Future research should investigate whether the results are also applicable to other regions in and outside Germany with other (socio-)cultural backgrounds. Patients from a variety of clinical departments were surveyed and there is no over-representation of a specific cohort of patients in our sample. We were able to reach a large number of men who would normally be incapable to take part in such research (due to age- or health-restrictions) by distributing the questionnaire in the patient rooms. Since our questionnaire was available in five different languages, we were able to recruit a large and diverse sample.

## Conclusion

This study surveyed the lifetime prevalence of violence and victimization and found that the victim-perpetrator group is the largest cohort of men within the violence spectrum. Our findings showed that due to the severity and extent of the experienced violence, this group reports the poorest health outcomes rendering it specifically vulnerable. These results provide important implications for practice and future research. As violence experience is associated with poor health outcomes and thus increased societal costs, effective intervention and prevention programs should be developed to either prevent future violence or to empower victims and perpetrators to identify and cope with maladaptive behaviors. Moreover, our findings highlight the need for healthcare professionals to build a comprehensive account of potential health-related outcomes when treating individuals who have been exposed to or engaged in violence. Many existing intervention and prevention programs treat either victims or perpetrators, but it may be of benefit to develop more comprehensive programs which focus on victimization and perpetration coincident. Moreover, to prevent negative health consequences of violence, prevention and/or intervention programs should ideally start before or during adolescence. Violence prevention should not only focus on reducing the type of violence that appears to be the problem at any given time but should also address other types of violence. Otherwise, there might only be a shift in the types of violence used as compensation or coping strategies. For future research it is of great relevance to investigate which type of victimization and/or perpetration is related to which consequences. Furthermore, to clarify the causal relationship between violence and health longitudinal research is necessary. Results could contribute to the development of targeted and individualized treatments and interventions.

## Data Availability Statement

The datasets presented in this article are not available because data sharing is prevented by ethical, privacy, or confidentiality matters. We do not have the ethical approval to share the data. Requests to access the datasets should be directed to DL, dleiding@ukaachen.de.

## Ethics Statement

The studies involving human participants were reviewed and approved by Committee for Clinical Research of the RWTH Aachen University Hospital. Written informed consent for participation was not required for this study in accordance with the national legislation and the institutional requirements.

## Author Contributions

UH, RB, RK, DL, and MS contributed to conception and design of the study. RB, FK, RK, and DL substantially contributed to the acquisition of data. DL and FK were responsible for data management. DL performed the statistical analyses and wrote the first draft of the manuscript. PH and AP contributed to the interpretation of the data and revising the manuscript critically for important intellectual content. All authors contributed to manuscript revision and final approval.

## Conflict of Interest

RB is employed by “Berufsförderungswerk Düren GmbH”. He was not employed there during the project period of the survey and evaluation of the study. The “Berufsförderungswerk Düren” was not involved in the study. The remaining authors declare that the research was conducted in the absence of any commercial or financial relationships that could be construed as a potential conflict of interest.

## Publisher’s Note

All claims expressed in this article are solely those of the authors and do not necessarily represent those of their affiliated organizations, or those of the publisher, the editors and the reviewers. Any product that may be evaluated in this article, or claim that may be made by its manufacturer, is not guaranteed or endorsed by the publisher.
