# Supplementary material for: Lifetime Prevalence of Victimization and Perpetration as Related to Men’s Health: Clinical Insights
Source: Front Psychol. 2022 Mar 15;13:762079. doi: 10.3389/fpsyg.2022.762079 (PMC8965285; doi:10.3389/fpsyg.2022.762079)
Supplement: Supplementary file 1 [file Table_1.DOCX]

Supplementary Material

**Appendix A**

Supplementary table 1 provides an overview of demographic information of the participants.

SUPPLEMENTARY TABLE 1. *Sociodemographic characteristics of the total sample with and without missing data on violence questions.*

| *Table 1.* Sociodemographic characteristics of the sample without missing data (N=4,601) and with missing data*(N=784) | | |
| --- | --- | --- |
|  | **No missing data (n=4,601)** | **Missing data on violence (n=784)** |
|  | N (Means ± SD) or (%) | |
| **Age** | n=4,108 | n=625 |
|  | 55.6 (±17.8) | 59.3 (±16.5) |
|  |  |  |
| **Country of birth** | n=4,399 | n=694 |
| Germany | 3,861 (87.8%) | 596 (85.9%) |
| Other Countries | 538 (12.2%) | 98 (14.1%) |
|  |  |  |
| **Educational degree** | n=4,539 | n=765 |
| No educational degree | 84 (1.9%) | 27 (3.5%) |
| Lower secondary school | 1,586 (34.9%) | 392 (51.2%) |
| Secondary school degree | 994 (21.9%) | 171 (22.4%) |
| European Baccalaureate | 776 (17.1%) | 86 (11.2%) |
| University degree | 1,099 (24.2%) | 89 (11.6%) |
|  |  |  |
| **Marital status** | n=4,424 | n=755 |
| Single | 853 (19.3%) | 112 (14.8%) |
| In a relationship | 372 (8.4%) | 52 (6.9%) |
| Married | 2,581 (58.3%) | 479 (63.4%) |
| Divorced/ lived separated | 428 (9.7%) | 66 (8.7%) |
| Widowed | 190 (4.3%) | 46 (6.1%) |
|  |  |  |
| **Employment status** | n=4,333 | n=695 |
| Full-time | 1,727 (39.9%) | 251 (36.1%) |
| Part-Time | 131 (3.0%) | 20 (2.9%) |
| Self-employment | 235 (5.4%) | 32 (4.6%) |
| Training courses | 94 (2.2%) | 7 (1.0%) |
| Retired | 1,695 (39.1%) | 315 (45.3%) |
| Unemployment benefits | 259 (6.0%) | 37 (5.3%) |
| Incapacitated for work | 192 (4.4%) | 33 (4.7%) |
|  |  |  |
| **Annual gross income** | n=3,565 | n=487 |
| 0-10.000 | 466 (13.1%) | 78 (16.0%) |
| 10.000-20.000 | 643 (18.0%) | 120 (24.6%) |
| 20.000-40.000 | 1,242 (34.8%) | 171 (35.1%) |
| 40.000-60.000 | 729 (20.4%) | 77 (15.8%) |
| >60.000 | 484 (13.6%) | 41 (8.4%) |

* of either exposure to violence or perpetrated violence.

**Appendix B**

We created higher-order composite scores to investigate general health and violence and performed a factor analysis to examine which variables can be combined. Results of the factor analysis revealed two factors: one (Comp_psych_) includes psychiatric health outcomes (depression, anxiety, suicidal thoughts, PTSD, and sleep disorders) and the other one (Comp_phys_) reflects physical health outcomes (impairment of the motor system, permanent disabilities, and chronic pain).

Results showed that the victim and the overlap group reported significantly more psychiatric symptoms/mental health problems compared to the groups of no violence and perpetrators (Comp_psych_). Regarding somatic problems, all groups with violence experiences (V, P and O) reported significantly more health problems compared to the group which has neither victimization nor perpetration (Comp_phys_). See supplementary figure 1.


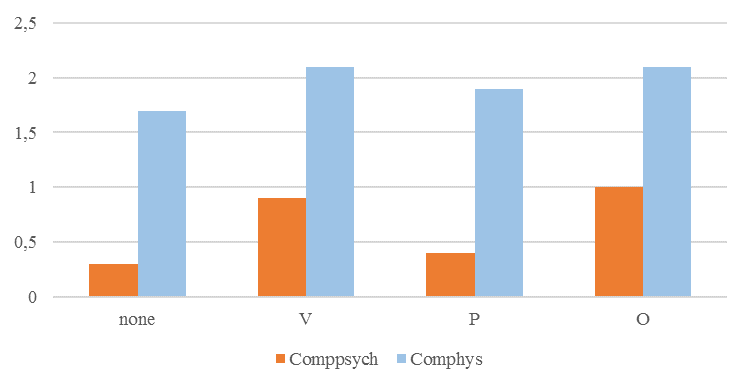


SUPPLEMENTARY FIGURE 1. *Composite scores of mental and physical health.*

Furthermore, we created higher-order composite scores to be able to make statements about general adverse health behaviors and violence. We performed a factor analysis, to examine which variables can be combined. Results of the factor analysis revealed 3 factors, of which one (Comp_legaldrugs_) includes legal drugs (use of nicotine, consumption of alcohol), the second one (Comp_illegaldrugs_) includes illegal drugs (psychedelic drugs, cannabis, ecstasy, cocaine and heroin) and the third one (Comp_riskbehavior_) other types of risky behaviors (increased sexual desire, risky sexual behavior, self-inflicted injuries, sedatives and gambling).

Results revealed, that the use of nicotine and the consumption was higher in the perpetrators and overlap group compared to the other groups. Regarding illegal drugs the overlap group had the highest composite score, the victims and the perpetrators did not differ and the group of no violence had the lowest score. Likewise for other risk factors, the victims and the perpetrators did not differ, the overlap group reported the most and the group of no violence the lowest risk behavior score. Also see supplementary figure 2.


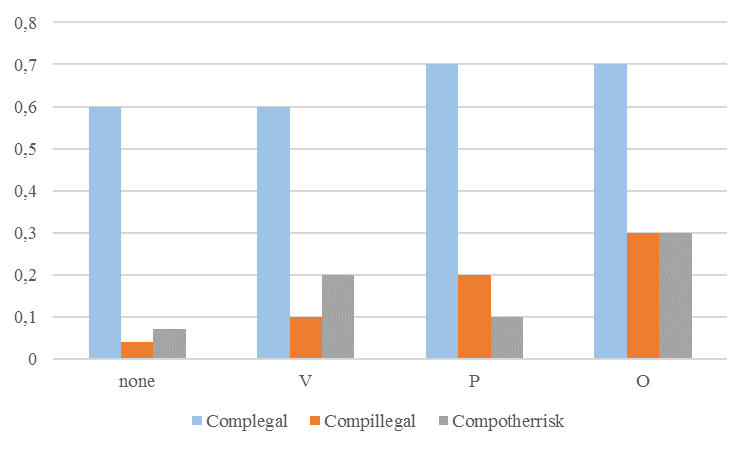


SUPPLEMENTARY FIGURE . *Composite scores of legal, illegal drugs and other risk behavior..*

**Appendix C**

Supplementary figure 3 shows by whom the victims of psychological violence in our data were perpetrated.

**
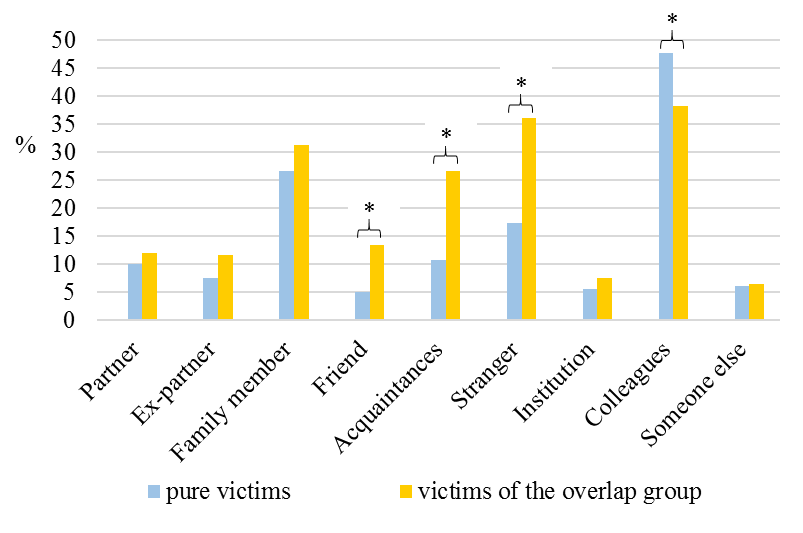
**

SUPPLEMENTARY FIGURE 3. *Perpetrators of exposure to psychological violence.*
